# Evidentiary Authority as a System: Johann Christoph Gatterer and the Collective Making of Historical Knowledge in the Eighteenth Century

**DOI:** 10.1002/bewi.2145

**Published:** 2025-07-02

**Authors:** André de Melo Araújo

**Affiliations:** ^1^ Department of History University of Brasília Brasília 70910‐900 Brazil

**Keywords:** diplomatics, early modern period, empiricism, genealogy, history of historiography, history of knowledge, visual evidence

## Abstract

How is historical evidence conveyed? How could an eighteenth‐century scholar vouch for the information stored on paper, drafted with the quill, and publicized in copperplate engravings or letterpress? In this article, I employ material and medial perspectives to reconstruct the multiple production stages of Johann Christoph Gatterer's *Historia genealogica dominorum Holzschuherorum* (1755) and, thereby, reveal how historical knowledge was shaped by the media that presented it. By focusing not only on the text but mainly on the engraved plates inserted within the pages of this work, I will reveal how, in the eighteenth century, historical knowledge was collectively achieved through complex scholarly, artistic, and editorial negotiations that encompassed issues of authorship and intellectual authority as well as disputes that occurred both in the making of visual evidence and the trading of authoritative editions. After exploring many drawn, handwritten, typeset, and engraved sources related to this editorial project, I argue that Gatterer's work relied on an information system based on the interplay between verbal and visual information and their relationship to the material evidence of the past. Moreover, I show how this system itself was shaped by the different media that it, in turn, used to reproduce historical evidence.

## 1 Introduction

How is historical evidence conveyed? This question captured the attention of Johann Christoph Gatterer (1727–1799), an eminent eighteenth‐century professor of history at the University of Göttingen, who sought to meticulously articulate a precise and methodical science in every scholarly work.^1^ At the end of his career, Gatterer was concerned about how historical arguments were put to paper with the quill and how they were worked out when finding their way to the printing press as even the best research could lose its authority if it were not carefully conveyed. In his eyes, this was the case with Abbot Gottfried Bessel's (1672–1749) *Chronicon Gotwicense*. Although Bessel's work, published in 1732, was a milestone in the German book market, particularly in the field of diplomatics, and contributed to the advancement of systematic criticism of historical documents, Gatterer dismissed its copperplate engravings as of little value for scientific purposes. According to him, one learns little from the charters reproduced in the printed volume since their format “was adapted to the narrow folio width of the book, […] giving a false idea of the format and overall external appearance of the originals.”^2^

When criticizing reproductions of historical evidence at the end of the eighteenth century, Gatterer was not alone. A few months after his critical evaluation began to circulate, the periodical *Der Neue Teutsche Merkur* published a text by the numismatist Karl August Böttiger (1760–1835) featuring the opinion that even the best engravings of coins could, at most, give just a general idea of the original artifacts they depict.^3^ Both evaluations make it clear that historical evidence was seen to be shaped by the media through which it was conveyed. How could an eighteenth‐century scholar vouch for the arguments stored on paper, drafted with the quill, and publicized in copperplate engravings or letterpress? In this paper, I employ material and medial perspectives to reconstruct the multiple production stages of an early modern illustrated book and, thereby, reveal how historical knowledge took shape through its presentation media. Given that Gatterer delivered his critique of Bessel's *Chronicon Gotwicense* from a professorial chair, I take a step back to examine to what extent the illustrated book, which had motivated his appointment as a professor of History at the University of Göttingen four decades earlier, might also be subject to a similar critique.

On October 16, 1755, Christoph Sigmund von Holzschuher (1729–1779) reported in his private correspondences that Gerlach Adolph Freiherr von Münchhausen (1688–1770), the first curator of the newly established University of Göttingen, had received the recently published *Historia genealogica dominorum Holzschuherorum* ^4^ so favorably that he offered Gatterer a well‐paid professorial post. In Göttingen, the young scholar was to dedicate his time “solely to history.”^5^ The book that had convinced Münchhausen of Gatterer's talent on historical subjects was subsequently commissioned by Christoph Sigmund's father, Karl Sigmund von Holzschuher (1687–1760), who, at that time, was the head of one of the oldest patrician families in the free imperial city of Nuremberg. On May 19, 1752, Karl Sigmund proposed that a genealogical history of the Holzschuher family should be compiled based on the documents collected so far.^6^ Following the footsteps of his ancestors, he had taken on the tradition of gathering genealogical data about his lineage. However, he believed that the continuation of this tradition in future generations should no longer rely on the laborious work of the quill. Rather, it should be made to reach a much broader audience as a product of the printing press. Gatterer was the local scholar commissioned to be in charge of this editorial enterprise.

When retrospectively evaluating the outcomes of this work, Gatterer, then professor in Göttingen, characterized his *Historia genealogica* as “an unfortunate middling of panegyric and history.”^7^ Indeed, a panegyrical tone characterized many early modern genealogies of noble families^8^ and was undoubtedly expected by the Holzschuhers. In 1755, the Holzschuhers could read in letterpress that the virtue of their family “is the same today as it has been for so many centuries,”^9^ according to the Latin words of Iohannes Christophorvs Gatterer. However, as the final product of the printing press was also to be perceived as a work of history, the information amassed in the volume needed to be buttressed by authoritative historical evidence. To this end, Karl Sigmund granted Gatterer access to the documents preserved by the family.

**Figure 1:**
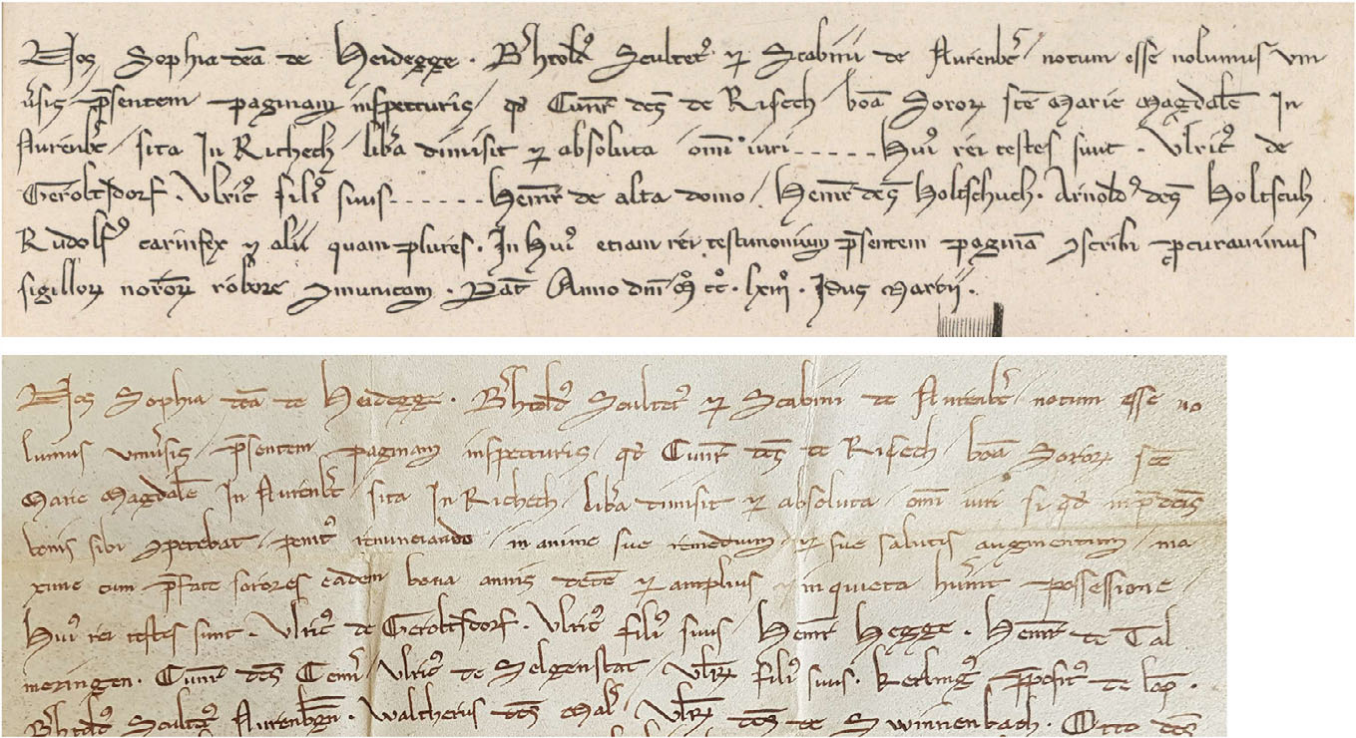
Above—Detail of Plate XIII: Gatterer 1755. Niedersächsische Staats‐ und Universitätsbibliothek Göttingen [henceforth SUB Göttingen], 2 H BAV II 3102. Below—Charter from 1263, March 15. Staatsarchiv Nürnberg, StAN Reichsstadt Nürnberg, Urkunden vor 1401 Nr. 33.

According to the young scholar engaged in this editorial project, “historical truth only deserves this venerable name when it is supported by authoritative testimony and evidence.”^10^ Gatterer was in search of both. Aware of Gatterer's intentions, Karl Sigmund convinced his family on May 18, 1753 that authoritative evidence of their noble past should also be disseminated to a broad readership. At this point, copperplate engravings were commissioned.^11^ Although some followed the early modern tradition of illustrating a printed volume with portraits and coats of arms of the portrayed family, the largest ones were designed to provide visual evidence of the historical information presented throughout the text. In the particular case of the charter from March 15, 1263, reproduced in plate XIII, the original is still extant and is now housed at the Nuremberg State Archives (**Figure** **1**).

As shown in Figure 1, the copperplate engraving does not reproduce the width of the original charter; it also breaks the lines of text in different positions and abbreviates its content in a manner very similar to that which, as we have seen, Gatterer himself would some decades later deplore in his review of the copperplate engravings printed for Bessel's *Chronicon Gotwicense*. Additionally, the format of the horizontal charters seems to have been adapted to the width of the printed media, giving once more “a false idea of the format and overall external appearance of the originals.”^12^ What was at stake when historical evidence was visualized within Gatterer's genealogical work? What might eighteenth‐century scholars and patricians have expected from the engraver commissioned to reproduce them in another media? How far could the printed image convey evidentiary authority and thereby underpin the arguments presented to the eyes of a large audience in letterpress?

Here, I argue that Gatterer's illustrated book, published in 1755, relied on an information system based on the interplay between verbal and visual information as well as their relationship to the (re)produced material evidence of the past. Moreover, I show, on the one hand, how this system was shaped by the different media through which it was produced and, on the other, how it reproduced historical evidence. By focusing not only on the text but mainly on the engraved plates inserted within the pages of the *Historia genealogica*, I will reveal, first, how, in the eighteenth century, historical knowledge was produced through complex scholarly, artistic, and editorial negotiations that encompassed issues of graphic and intellectual authorship and intellectual authority as well as disputes that occurred both in the making of visual evidence and the trading of authoritative editions. Second, I will show how nobles, scholars, amanuenses, printers, and artists pooled their efforts to provide evidentiary authority for the arguments reproduced in Gatterer's genealogical work. By exploring how information was (re)produced, authenticated, and shared, my analysis focuses on eighteenth‐century historical writing through the lens of contemporary research on the history of knowledge. Therefore, instead of just stressing the political value that genealogies of noble families had in the Early Modern period, I underscore how historical knowledge was construed and shaped by collectively performed practices in this period.

## 2 Authoritative Work and Empirical Testimony

In the eighteenth century, diplomatics had already been established as the branch of knowledge in charge of distinguishing forgeries from authentic written documents. Together with genealogy, chronology, and heraldry, it constituted the core of the early modern auxiliary sciences of history. In 1756, one year after the publication of the *Historia genealogica*, Gatterer taught his first course on diplomatics in Nuremberg. He was acquainted with the methodological procedures of this auxiliary science after following the lectures of Johann Heumann (1711–1760), who relied on his extensive collection of seals to solve problems of forgeries in legal disputes.^13^ However, when Heumann could not count on having original seals for his teaching activities, he relied on reproductions.^14^

In early modern collections of artifacts, the absence of originals in a series was the rule.^15^ At that time, different techniques were developed within antiquarian circles to make copies of collectibles, thereby overcoming gaps in a collection. It is for this reason that “authoritative images certified by authoritative observers” were in circulation among scholars. They were “made with the intent not only of depicting the object of scientific inquiry but also of replacing it,” as argued by Lorraine Daston.^16^ Therefore, originals and reproductions customarily constituted an essential part of a collection used for teaching purposes in the 1700s. However, not every reproduction was made by looking at the original artifact they replaced and depicted. Instead, they were frequently the graphic expression of a larger information chain fostered by the editorial market.

**Figure 2:**
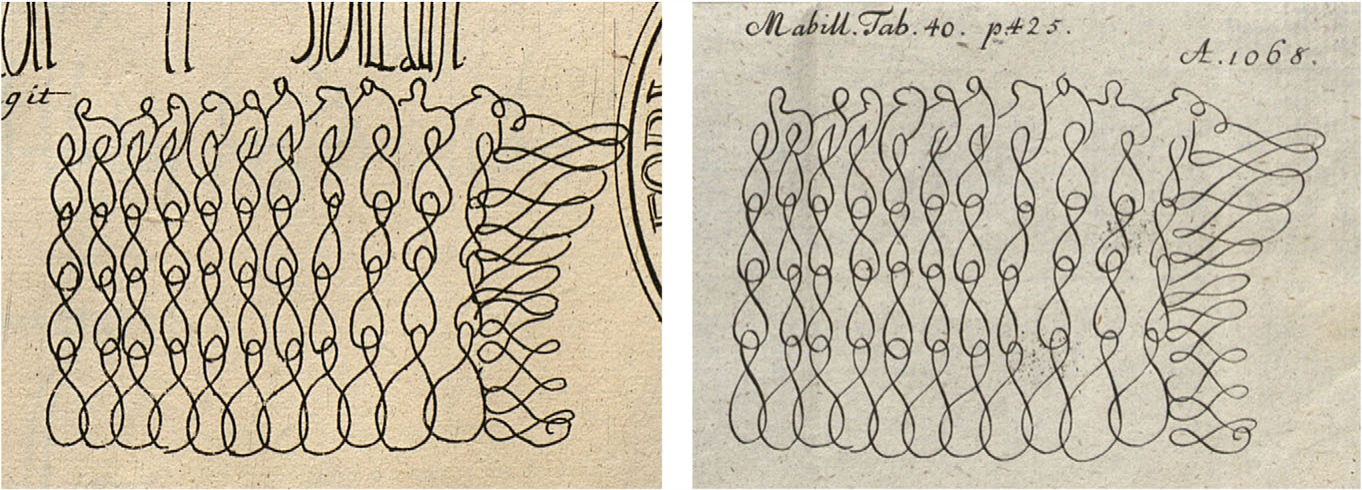
On the left—Detail of Plate XL: Mabillon 1681, on 425. Landesbibliothek Oldenburg, LIT V 3 216,1. On the right—Detail of Plate VII: Gatterer 1798. SUB Göttingen, 8 H SUBS 2018.

In 1745, Gatterer's academic mentor published a commentary on diplomatics.^17^ The title page of Heumann's work shows a thematic and a visual affinity to the book's point of reference: Jean Mabillon's (1632–1707) *De Re Diplomatica*. The new German title displays a copy of the frontispiece that figured in many French editions of Mabillon's study, first published in 1681.^18^ As is widely known in the history of historiography, Mabillon was involved in disputes over the authenticity of documents in the seventeenth century.^19^ His method for authenticating medieval documents was disseminated with the help of copperplate engravings, which, in turn, were based on specimens from early manuscripts collected by his acquaintances across Europe.^20^ “Above all, he used engraving, the central, and powerful, illustrative technology of his time, to present the evidence to the reader,” says Anthony Grafton. “No one had examined and illustrated so wide a range of manuscripts as Mabillon did. Inaccurate though his plates often were in detail, their impact was explosive,”^21^ reaching far beyond the French borders. In the German territories, for instance, Mabillon is the source for the Carolingian script in Heumann's work.^22^ At the end of the century, the image printed in France was also replicated in Gatterer's *Abriss der Diplomatik* (1798), a handbook on diplomatics published at the end of his life,^23^ as seen in **Figure** **2**. It displays particular graphic elements (*Rekognitionszeichen*) featured in a French charter issued in 1068 during Philip I's reign (1060–1108). Within the framework of the *semiotica notarialis*—a term introduced in Gatterer's seminal work on diplomatics that refers to the analysis of seals and other graphic signs crucial for authenticating written documents^24^—these external features of a medieval charter were paramount for dating and distinguishing an authentic written document (Figure 2).

The engraving found within the pages of this late‐eighteenth‐century German handbook on diplomatics was made from another image that was inserted in an authoritative work on the same subject, instead of being copied from the original historical document it depicts. In this case, the visual information printed on the page—as it is also the case with many other examples displayed in Gatterer's *Abriss der Diplomatik* and previously in his *Elementa Artis Diplomaticae Universalis*—does not show what the engraver saw; rather, it reproduced information that early modern communities of scholars could rely on and must have been closely acquainted with, such as Mabillon's printed work. These chains of visual information, characteristic of early modern collective and cumulative empiricism, warrant further examination from a broader book historical perspective.

The French diplomatic and antiquarian tradition was well received in the German‐speaking territories due to the publications of Heumann and Gatterer as well as to a mid‐eighteenth‐century translation of Bernard de Montfaucon's (1655–1741) lavishly illustrated volumes on Greek and Roman antiquities. The text of the German edition of Montfaucon's work was printed in 1757 by Johann Joseph Fleischmann,^25^ the same printer who was in charge, just 2 years before, of publishing Gatterer's *Historia genealogica*. In both cases, the printer prepared the volumes to accommodate multiple copperplate engravings. Regarding Montfaucon's German edition, they were praised as “imprints of the best originals.”^26^ Yet, what the titles printed by Fleischmann in 1755 and 1757 further reveal is, first, that they were the product of collaborative work and, second, that how engravings were replicated and authentic remains of the past were depicted in copperplates mattered for the arguments printed in letterpress.

**Figure 3:**
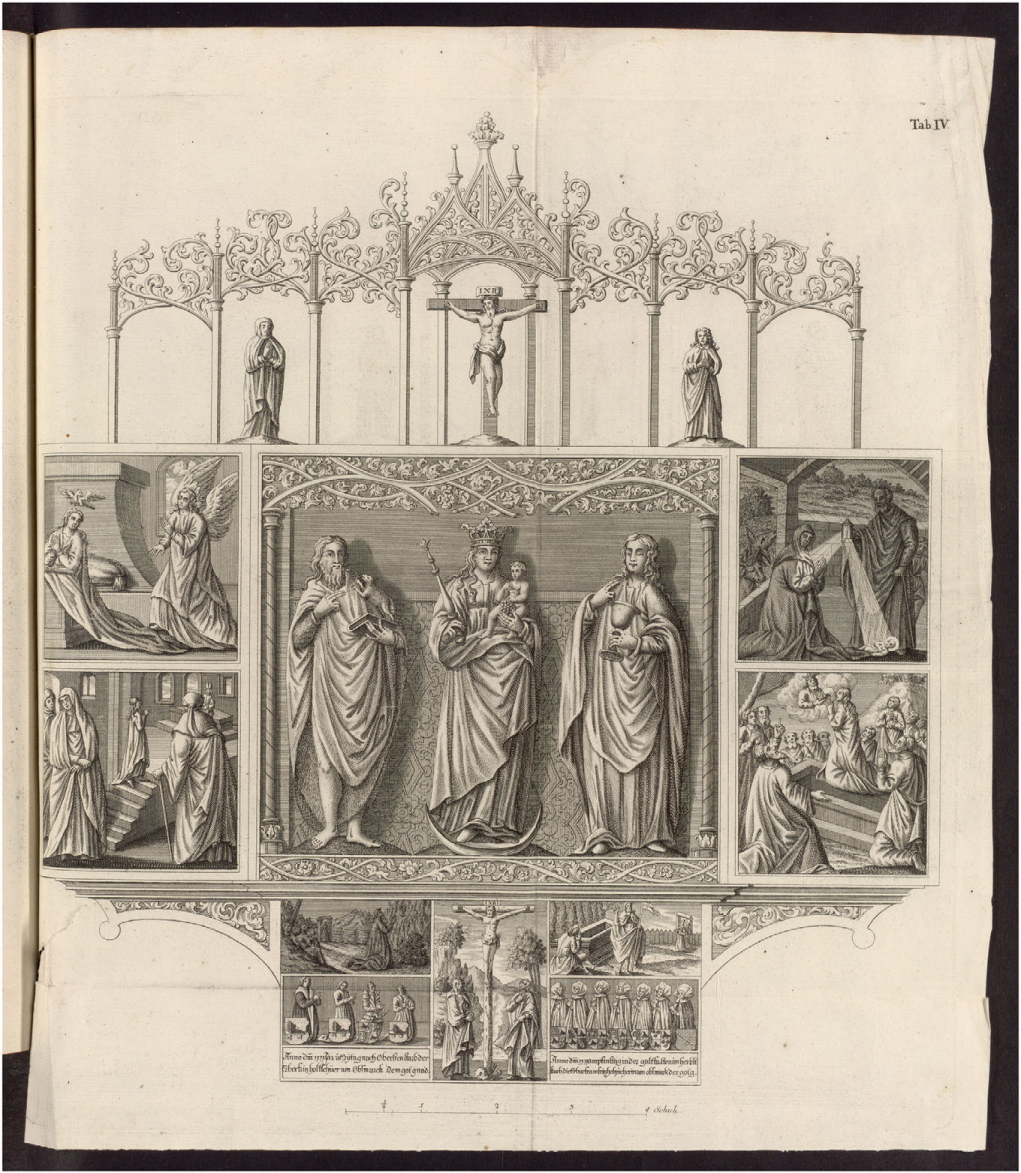
Plate IV: Gatterer 1755. SUB Göttingen, 2 H BAV II 3102.

In the preface to the *Historia genealogica*, Gatterer describes the engraved frontispiece in the following way: “The central section displays both the older and more recent insignia of the Holzschuher family, adorned with symbols of the toga, a military cloak, a sword, and two crosses hanging from ribbons, representing the knightly honor that distinguished them.”^27^ Here, the verbal descriptions of the iconographical content of an engraving are primarily aimed at explaining a visual message most readers would find unintelligible. Almost all other engravings, however, needed to convey a more straightforward message, for “the testimonial force of artifacts”^28^ they strove to depict was required to provide trustworthy evidence of the noble past of the family. Such artifacts included, besides charters, pieces of tapestry, stained‐glass windows, goblets, and funerary hatchments, all of them carefully depicted on plates specially designed for the book, as, for instance, a wooden altarpiece commissioned by the Holzschuhers in the sixteenth century (**Figure** **3**).^29^

**Figure 4:**
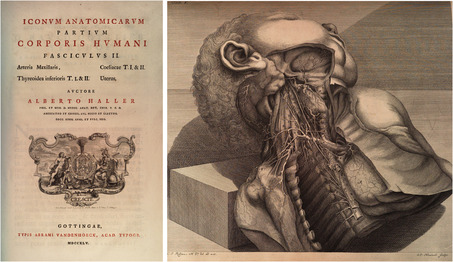
On the left—Title Page: Haller 1745. On the right—Plate: Haller 1745. Universidad Complutense de Madrid, HathiTrust.

Although this plate remained unsigned, it was almost certainly the work of Georg Daniel Heumann (1691–1759), who had engraved another altar depicted in the previous plate of the same book. Before moving back to Nuremberg in the early 1750s, Heumann engraved portraits of several professors at the University of Göttingen and images of the university buildings.^30^ In Göttingen, he was responsible for engraving the plates depicting the human body in the works of Albrecht von Haller (1708–1777), a physiologist who was “reputed to have prepared specimens of some anatomical region as many as fifty times to make sure that the artist had a representative rather than anomalous model, displayed in characteristic circumstances.”^31^ Haller's *Icones Anatomicae* (1743–1756) testify to the eighteenth‐century experimental culture.^32^ Heumann's engravings of the human body were based on a drawing by Christian Jeremias Rollin (1703–1781), who assures us in the inscription in the lower part of the image that he had observed the body depicted.^33^ Albrecht von Haller vouched for Rollin's drawings,^34^ stressing that the latter were certified by authoritative scholars and talented observers (**Figure** **4**).^35^

As a professor of anatomy and surgery at the University of Göttingen, Haller was also greatly interested in the anatomical structures of other animals; he wrote a foreword for the *Historia Naturalis Ranarum Nostratium*. This work would soon be praised for its many hand‐colored copper engravings of frogs and toads native to the Nuremberg region. In the foreword, Haller stated that the ensuing plates were designed only after careful and close observation of nature.^36^ This book was published in Nuremberg in 1758 by Johann Joseph Fleischmann, who was also the printer of Gatterer's genealogical history. In the same year, Fleischmann also published William Smellie's *Tabulae Anatomicae*,^37^ a book of instruction for midwives, whose illustrations were engraved and printed by Johann Michael Seligmann (1720–1762).^38^ This artist additionally engraved some of the plates commissioned for the genealogical history of the Holzschuher family.

Collectively, these works printed in the mid‐eighteenth century reveal two important aspects. First, collaborations and networking between printers and artists were quite common in the early modern German book market. Second, the images produced within this collaborative network of artists and printers for scientific publications emphasized that the information (re)produced in the engravings stemmed from direct empirical observation of nature and, similarly, from the material remains of the past. The reliability of empirical testimony was a crucial element in authenticating both natural scientific and humanities materials. Yet, to better understand how evidentiary authority was conveyed in historical works, we must take a step back to explore the production of historical knowledge. With this aim in mind, I will demonstrate in the following sections that Gatterer's conception of evidentiary authority was conceived within the framework of an information system he first developed in the 1750s. Accordingly, we must look beyond the printed plates to get a comprehensive overview of how this system was designed and managed.

## 3 Material Evidence of a Collective Enterprise

On the engraving of the altarpiece to St. Johannis in Nuremberg commissioned by the Holzschuhers (Figure 3), the artist made a mistake that could put the accuracy of the information printed in the book at risk. In this case, the information concerned the death year of Friedrich Holzschuher's wife. Instead of the year 1511, the plate should have displayed the year 1521 in the inscription located in the lower part of the right side of the altarpiece. In the preface to the *Historia genealogica*, Gatterer made the following textual intervention: “In the later inscription on this altar, which marks the year of death of Friedrich Holzschuher's wife, the year 1511 was mistakenly inscribed instead of 1521 due to an error by the sculptor. See below, p. 260.”^39^

Whereas the preface indicates the mistake printed on the plate, it also serves to reassure the accuracy of the information printed in letterpress in the main text of the work. The correct information was available in the section of the book entitled *Pars Specialis Historiae Holzschuherianae*, in which the dates related to the history of the Holzschuher lineage are presented, including the years in which Friedrich Holzschuher and his wife each died. In turn, the textual passage makes further reference to a *Codex diplomatum*.^40^ This indication is part of what Martin Gierl calls “reference system of graphic evidence.”^41^ In this case, the system was designed by Gatterer to enable the readers to navigate the book and ensure the accuracy of the information displayed on its pages.

In addition to the prefatory pages and the main text, the work includes exhaustive transcriptions of charters and other historical documents in a section entitled *Codex diplomatum et documentorum […]*, as well as the engraved plates. Indeed, the volume features more than 300 transcribed documents, which were subsequently printed in letterpress. Some were partially depicted in the 39 copperplate engravings produced between 1747 and 1754 by at least five different engravers. Whereas the *Codex* was retrospectively evaluated by Gatterer as “made with careful choice and correctness,”^42^ since he could proudly vouch for every letter of the transcriptions,^43^ the copperplate engravings were appraised as “useful, faithful to the originals,” and “mostly well printed.”^44^ Here, however, he wondered how historical evidence could be faithfully reproduced when the artist's empirical testimony was not a sufficient safeguard against errors, as demonstrated in the case of the altarpiece.

Seeing how historical knowledge was shaped by the hands—and not just by the minds—of those whose names figured on the title page of an early modern book is often a difficult task. Apart from the fact that a wide range of handicrafts was always assembled to produce a book in an early modern printing shop, the variety of individual hands performing relatively distinct roles in generating the manuscript of the book we are considering here does testify to the collective nature of this particular editorial enterprise. Since the manuscript of the *Historia genealogica* is still preserved,^45^ we can trace the various contributors who provided information for the reference system designed and managed by Gatterer.

According to the Holzschuher family's archival registers, Gatterer had completed the work by May 31, 1754. At this point, he “had written 176 leaves, but the *Codex diplomaticus* consists of [a further] 331 leaves,” which were still under his scrutiny for corrections.^46^ The whole manuscript is a bifolium formed from different full sheets of paper folded in half.^47^ Its first block of text—corresponding to the Latin text printed between pages a2^r^ and e1^v^ in the published edition—was written on slightly smaller sheets of paper. The main text begins with another set of sheets and features distinct handwriting compared to the one in the first quire. This finding raises questions regarding the graphic authorship of the entire manuscript.

Gatterer's handwriting^48^ is featured in several places: the first quire; the portions of text corresponding to the letter to the reader; the very first page of the main part of the book. It can also be found in all genealogical trees and short notes written in the outer margin of the text. A second person was in charge of the transcriptions of historical documents in German—such as the one inserted between the pages of the text addressed to the *Lectori Benevolo*—a third for the transcriptions of charters and other historical documents in Latin, and a fourth was responsible for some corrections throughout the text and signing the break in the typeset page with a red pencil, as was the usual practice in a printing shop. Further handwriting is also featured in the *Pars Specialis* and the Index.

Gatterer inserted many marginal notes throughout the manuscript. In these notes, he frequently made corrections, amendments, and revisions to the text, in addition to introducing numbers above nearly every transcription of documents connected to the Holzschuher's history. Through such marks, he was able to arrange the transcriptions chronologically and manage their flow. The latter was particularly important given that many charters were transcribed in different paper stocks by several different contributors, including Gatterer's own students^49^ and an amanuensis working for the Holzschuher family.

**Figure 5:**
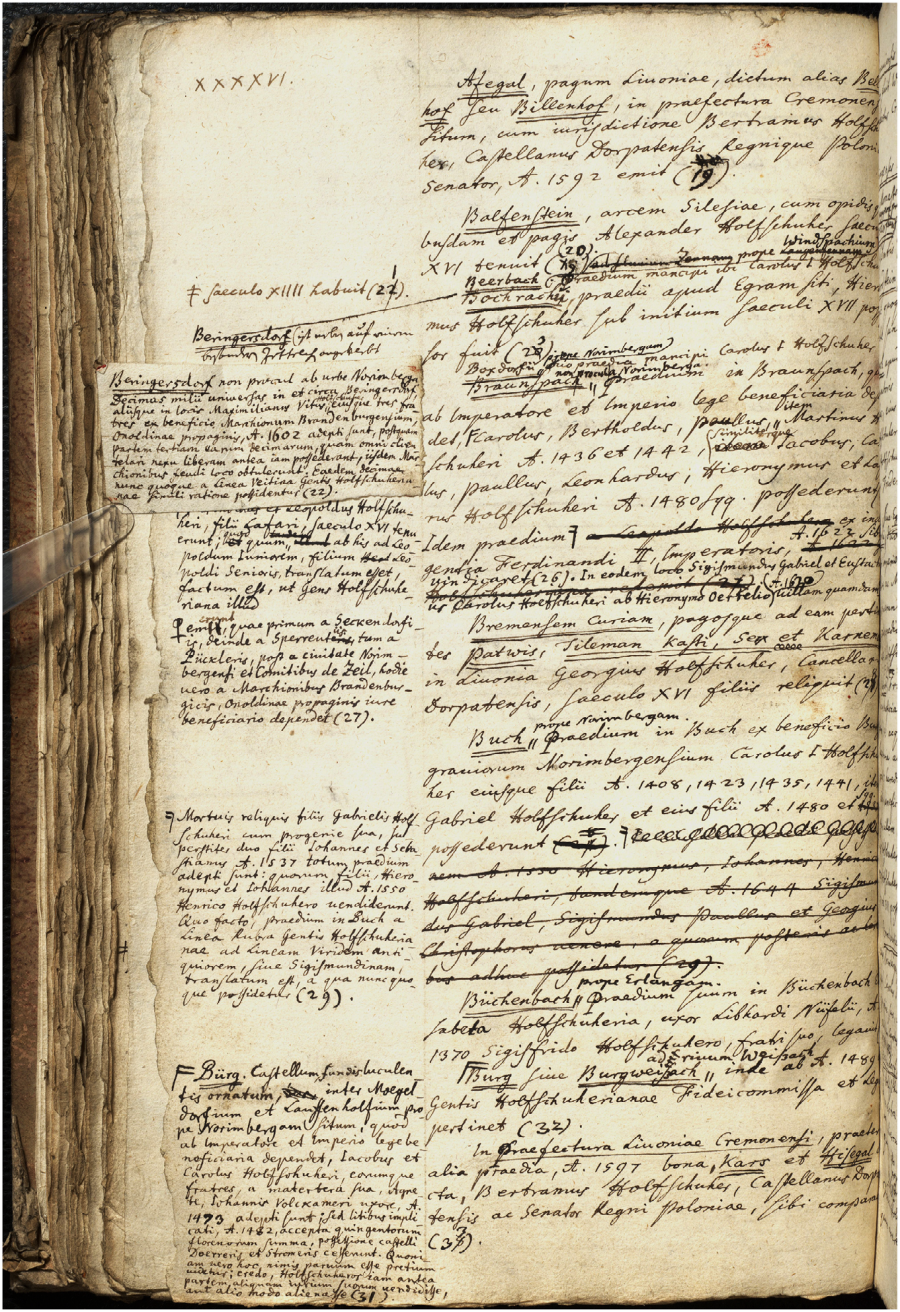
The handmade design of the reference system. Johannes Christophorus Gattererus, *Historia Genealogica Dominorum Holzschuherorum*, c. 1755. Bibliothek des Germanischen Nationalmuseums, Nürnberg, Hs 28 889.

As various hands collaborated in the manuscript's production, it is not surprising to occasionally find unmarked spaces left on the page for future contributions. These loose sheets of paper were not necessarily filled in following the order that they would appear in the printed book but were prepared in a way that could later be easily assembled in a different sequence. Similar to the approach that Matthew Daniel Eddy has recently taken to defining and analyzing many early modern notebooks, the manuscript generated and supervised by Gatterer can also be defined as “a specific kind of media technology, one that possessed interchangeable paper components and one that could be assembled and used with combinatorial patterns designed to impart and organize knowledge”^50^ anew. In addition to some interchangeable, loose‐leaf bifolia, the manuscript also contains handwritten notes on slips of paper pasted onto the sheets (**Figure** **5**). These strips of paper were a crucial component of the information system devised by Gatterer.

**Figure 6:**
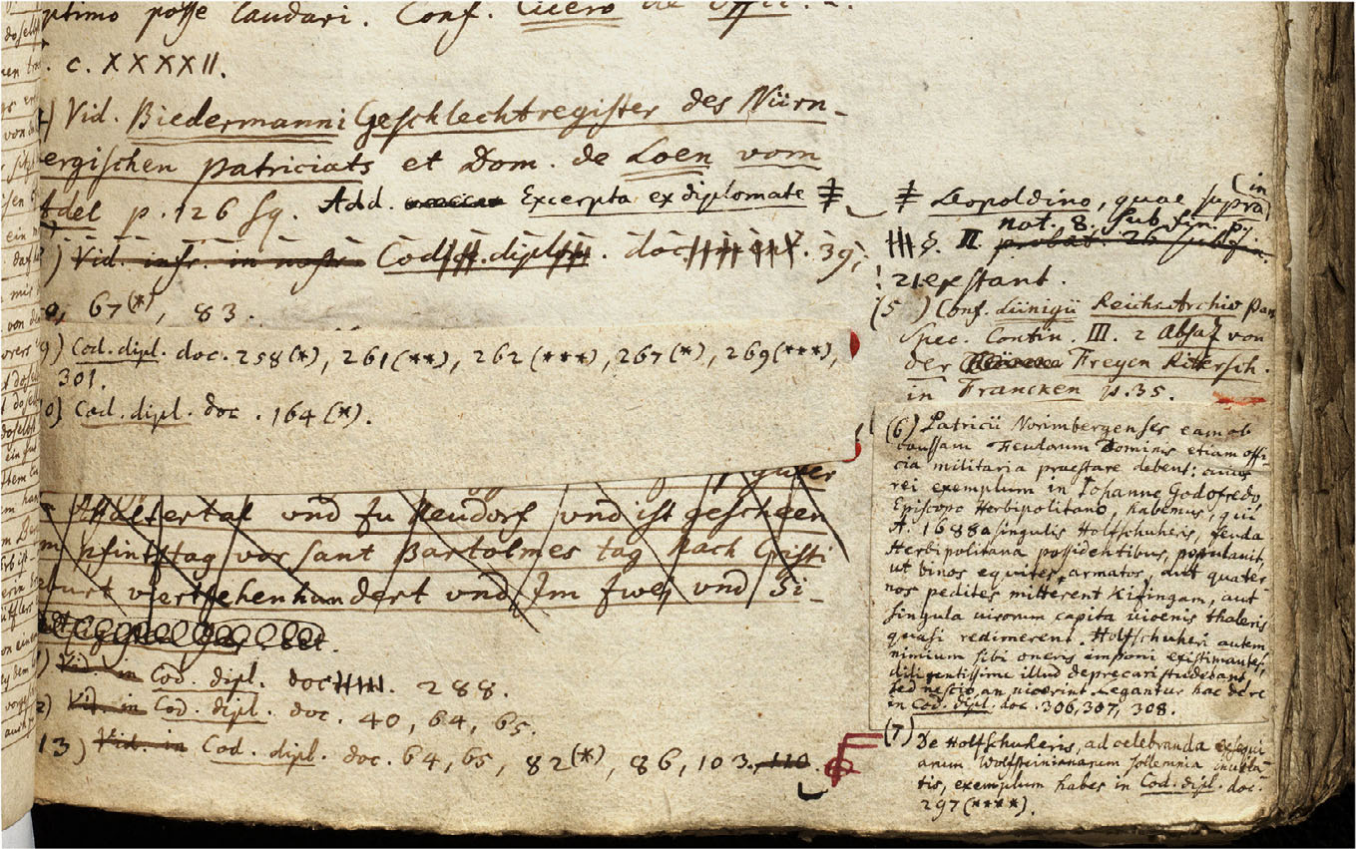
Johannes Christophorus Gattererus, *Historia Genealogica Dominorum Holzschuherorum*, c. 1755. Bibliothek des Germanischen Nationalmuseums, Nürnberg, Hs 28 889.

Among other things, this system allowed Gatterer to correct mistakes made by the engravers, such as the one mentioned in the depiction of an altarpiece. Here, he used slips of paper to both correct the error and refer to other parts of the books where the correct year of death for Friedrich Holszchuher's wife could be found. Notes such as this were collected and, in turn, created the internal reference system by connecting different documents through the numbers Gatterer inserted by hand (**Figure** **6**).

His working method made cross‐referencing possible for other passages or documents transcribed in the manuscript. Ann Blair has characterized this as a method of managing scholarly information. As she notes, manuscript notes on paper slips were commonly “used in the sixteenth century to distribute information within a complex organizational scheme, whether alphabetical or systematic.”^51^ While the corrections and additions made on strips of paper and in the margins reveal how Gatterer designed and used an information system, they also provide insight into additional practices underpinning the work published in 1755.

**Figure 7:**
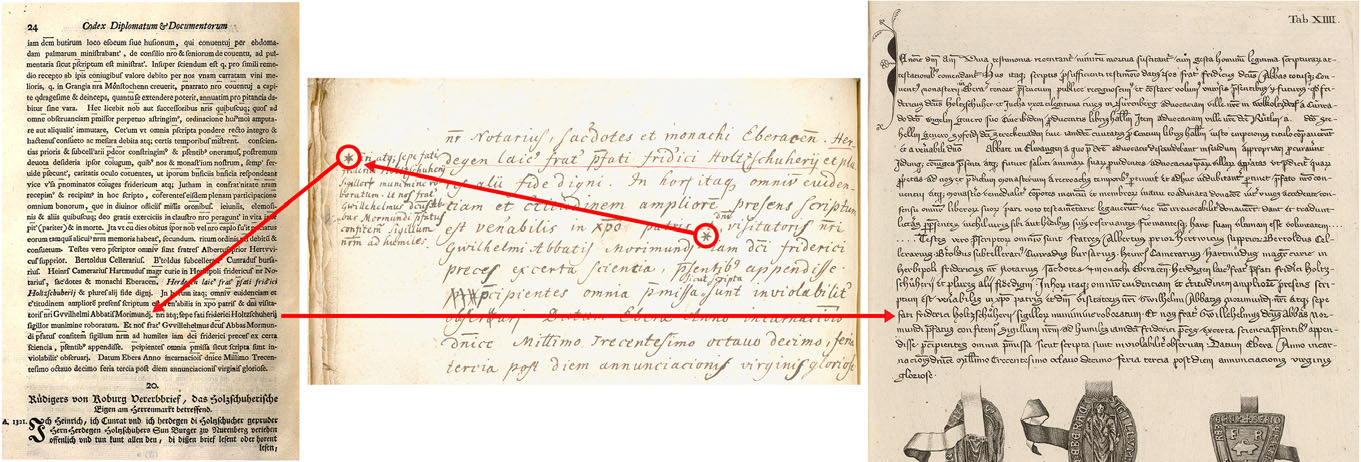
On the left—Gatterer 1755, on 24. Staats‐ und Stadtbibliothek Augsburg, 2 H 126. At the center—Johannes Christophorus Gattererus, *Historia Genealogica Dominorum Holzschuherorum*, c. 1755. Bibliothek des Germanischen Nationalmuseums, Nürnberg, Hs 28 889. On the right—Detail of Plate XIV: Gatterer 1755. SUB Göttingen, 2 H BAV II 3102.

The person in charge of transcribing a fourteenth‐century charter omitted a passage of its original text. The missing passage was included at the margin of the manuscript so that the full information would appear in the printed edition (**Figure** **7**). This charter is also reproduced in the book through a corresponding engraving. The engraving of this passage, in turn, presents the entire textual information of the charter it depicts (Figure 7), so neither the incomplete transcription of the manuscript nor its amended version was likely the source of information for the engraver, who worked on the plate in 1754, whereas the leaves corresponding to the transcriptions were in the hands of Gatterer for corrections.^52^

This case demonstrates that the information system envisioned by Gatterer was not restricted to what was recorded with a quill in the manuscript. Instead, it was supplemented by additional earlier works, which may have informed, at the very least, the artist tasked with reproducing what he had directly observed. It is to these earlier works that we now turn.

## 4 (Re)producing Evidence of a Noble Past

The Holzschuher family had a long tradition of compiling evidence of its noble past. Markus Friedrich argues that, throughout the Early Modern period, “genealogical information was a vital political resource, and so it was both therefore consciously generated and jealously guarded.” Within the Holy Roman Empire, the “most important manifestation of patrician genealogical culture”^53^ were genealogical books, such as the volume compiled by Lazarus Holzschuher (1487–1523) at the beginning of the sixteenth century, later bound in red velvet.^54^

Although the information recorded by hand may have been crucial, not all family records were passed down to subsequent generations with a lavish binding. Additional handwritten books and unbound sheets of paper containing genealogical and archival information about the Holzschuher family have also been preserved. These include a list of all male family members alive in 1511, a chronicle begun in 1542 with several seventeenth‐century additions, at least four eighteenth‐century collections of sources and document transcriptions related to the Holzschuhers, as well as a carefully compiled historical source for the family, held together by just a few stitches.^55^ The first quire of this collection of transcribed letters, charters, and further historical documents was likely penned by Veit Holzschuher around 1693, while the subsequent quires, larger in size, date from the next few years. Enclosed in this unbound volume is a single sheet of paper handwritten by Karl Sigmund Holzschuher. Given the variety of additions in the manuscript, the genealogical information appears to have been in continuous use by family members over the centuries. Other products of this tradition include two very similar copies, made by the same hand, of a 1736 genealogical book.^56^ Apart from the fact that the *Pars Specialis Historiae Holzschuerianae* of the 1755 printed work follows the genealogical and heraldic descriptions of the Holzschuhers as presented in this lavishly colored pair of manuscripts, both copies can also be connected with further records of the family through material means.

The paper used in both copies of this genealogical book displays a watermark that can also be found on the paper used in many transcriptions of documents included in the manuscript of Gatterer's *Historia genealogica*, as well as in the private correspondence of Karl Sigmund's son, Christoph Sigmund Holzschuher, between 1754 and 1755. Both the correspondences and the transcriptions were penned by the amanuensis previously mentioned, who worked for the Holzschuhers. His handwriting is also featured in another collection of 190 charters, partially transcribed and assembled around 1750.^57^ At this time, the head of the family was deeply concerned with managing this and another set of loose sheets containing transcriptions of various documents. It is for this reason that Karl Sigmund left testimony of his organizational intent by designing an index of the material transcribed and writing down a first draft of a *Genealogia Holzchueriana*.^58^ Indeed, exploring how the 1755 printed book might provide evidentiary authority for the family's noble past brings us to another set of transcriptions predominantly penned by Karl Sigmund Holzschuher himself.^59^

**Figure 8:**
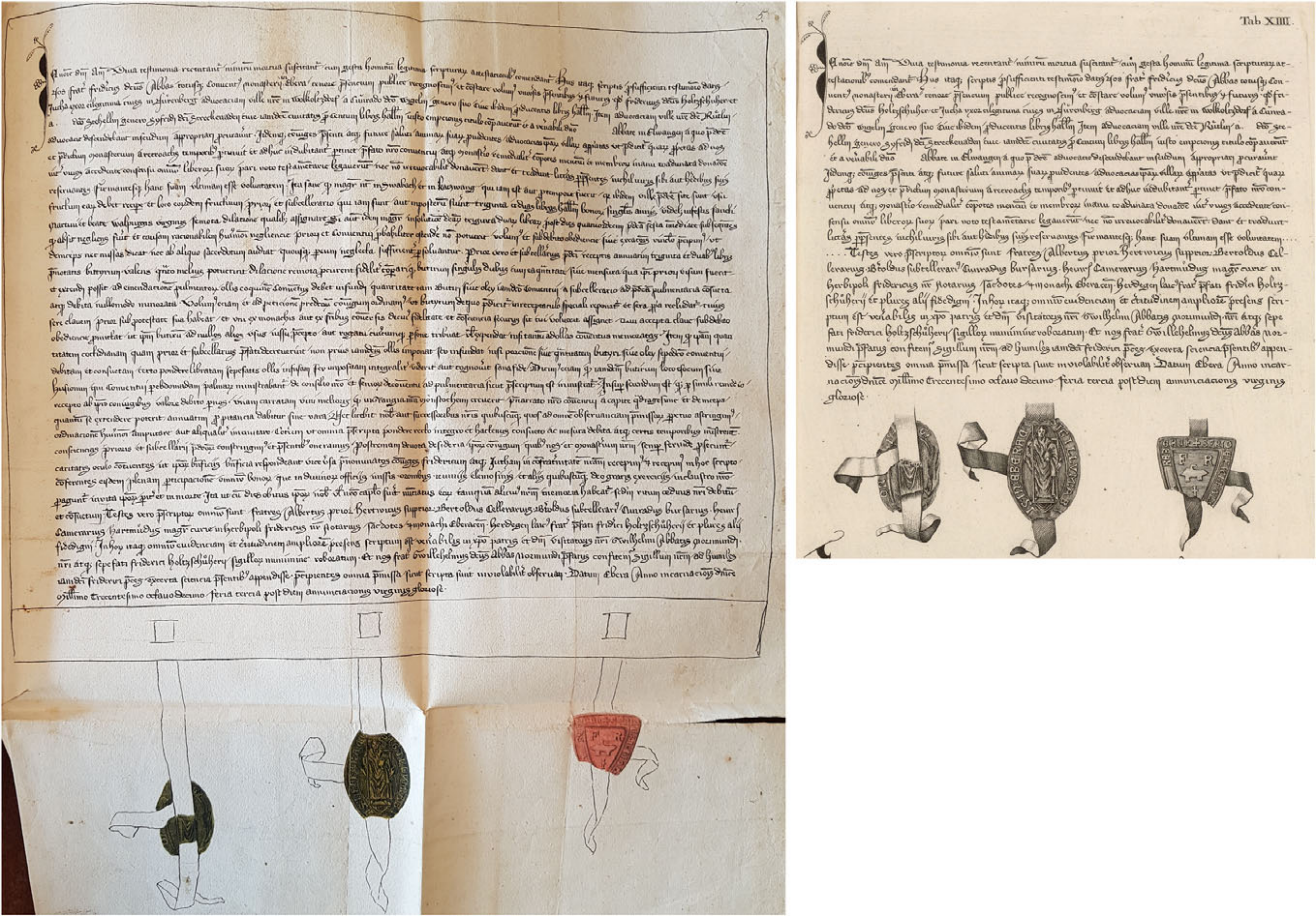
On the left—Drawing: *Anhang zur Holzschuherischen Historie […].* Historisches Archiv des Germanischen Nationalmuseums, Nürnberg, RS‐NBG 18‐Holzschuher‐15. On the right—Detail of Plate XIV: Gatterer 1755. SUB Göttingen, 2 H BAV II 3102.

In this set of handwritten bifolia, there are drawings of the charters later reproduced on plate XIV of the *Historia genealogica*. As shown in the previous section, the textual content of a particular charter dated 1318 was transcribed and amended in the book's manuscript before being typeset in the printing shop (**Figure** **7**). Yet, as expected from pictorial media, the drawing preserved in this group of handwritten bifolia displays the external appearance of the same charter in addition to reproducing its entire textual content. In contrast, the corresponding engraving commissioned by Karl Sigmund shows an abridged version of the text in which the external appearance of the medieval charter is altered, for instance, by inserting different line breaks (**Figure** **8**).

Close to this particular drawing, there are further reproductions of other charters engraved not only on plate XIV but also on the previous one. They now deserve a detailed discussion, as a closer look will help us unravel how historical knowledge took shape through the media that presented it.

**Figure 9:**
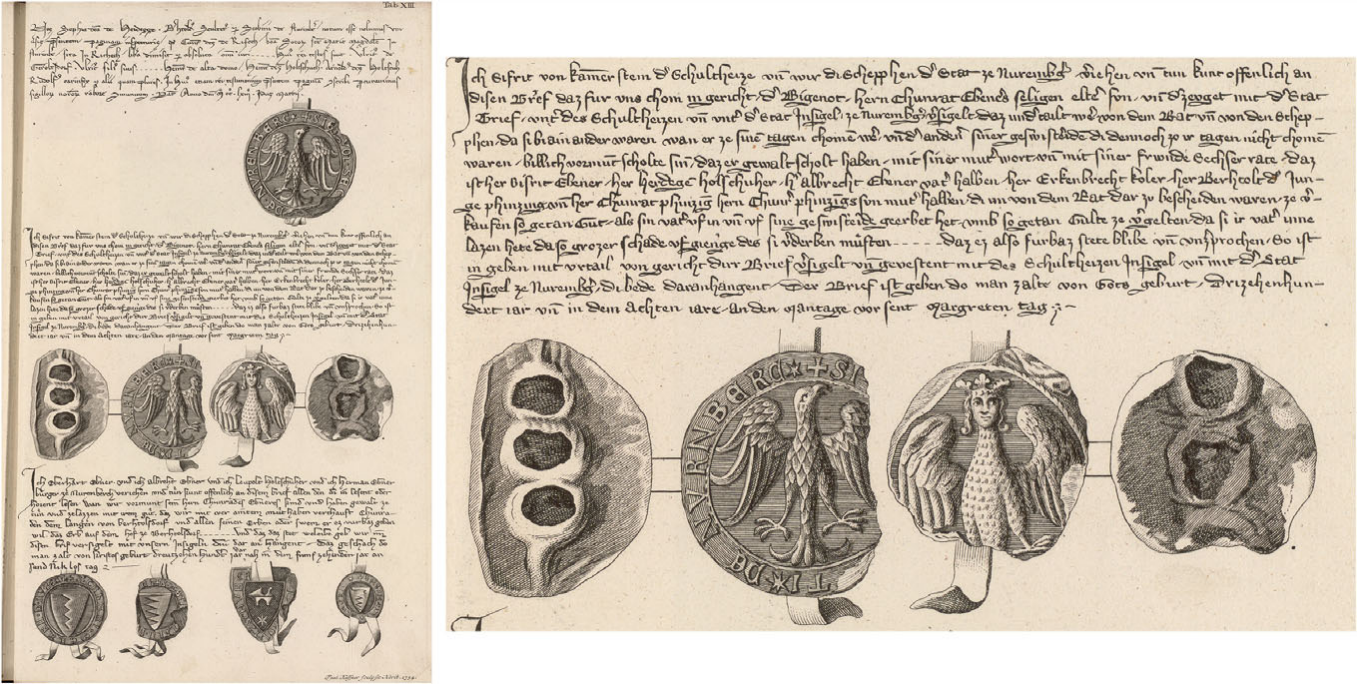
On the left—Plate XIII. On the right—Detail of Plate XIII: Gatterer 1755. SUB Göttingen, 2 H BAV II 3102.

The central portion of plate XIII depicts a document dated from 1308. It corresponds to the transcription number 16a printed in letterpress in the work, as indicated in the preface to the reader.^60^ As was customary in the hand‐press period, engravings printed separately on an intaglio press were added to a volume by the bookbinder, often inserted between the quires or at the end of the main text. Plate XIII, printed on a larger sheet of paper than those used for the main text, was partially folded and thought to be inserted after the pages printed mainly in letterpress. This plate portrays the handwritten form of the original document and the seals through which its authenticity could be assured (**Figure** **9**).

**Figure 10:**
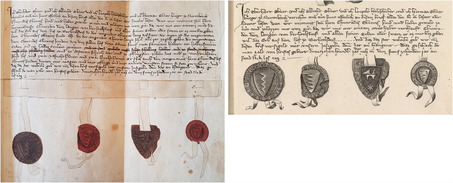
On the left—Drawing: *Anhang zur Holzschuherischen Historie […]*. Historisches Archiv des Germanischen Nationalmuseums, Nürnberg, RS‐NBG 18‐Holzschuher‐15. On the right—Detail of Plate XIII: Gatterer 1755. SUB Göttingen, 2 H BAV II 3102.

The plate was engraved in Nuremberg by Paul Küffner (1713–1786) in 1754. A few years later, he produced several copperplate engravings for the calligrapher Johann Christoph Albrecht (1710–1777), who had at that point authored manuals on the art of writing.^61^ From a broader perspective, engraved scripts constituted an essential component of print culture in the eighteenth century. They were common features not only in printed volumes on the art of diplomatics—such the publications of Mabillon and Gatterer—but also in pedagogical and antiquarian works as well as collections of letters. Engraved scripts were intended to convey to readers the impression that the engraver observed the authentic manuscript they depicted, thereby underscoring the reliability of their empirical testimony. Given that the primary purpose of the *Historia genealogica* was to familiarize a broader audience with the noble past of the Holzschuher family, the engravings commissioned by Karl Sigmund should convey evidentiary authority. Such a task, however, was not always carried out successfully from the perspective of late‐eighteenth‐century auxiliary sciences of history. For example, a comparison between the external appearance of a charter drawn in the bifolia, which was primarily written by Karl Sigmund, and the engraving printed in 1754 (**Figure** **10**) reveals that Küffner, rather than simply reproducing the drawings in another medium, made several changes. He omitted information and altered the format of the depicted charter when creating an abridged version of its text.

Abbreviating graphical and textual records of charters was a widespread practice among engravers in the Early Modern period, as long as the engravings displayed the visual signs in a way that assured the authenticity of the depicted artifacts.^62^ For this reason, in the preface to the work published in 1755, Gatterer informed his readership that the documents related to the history of the Holzschuher family were presented both in full and in excerpted form.^63^ Both forms would thoroughly fulfill the expectations of the commissioner, Karl Sigmund Holzschuher, but not always those of Gatterer, the late‐eighteenth‐century professor of history.

Since the *Historia genealogica* was a collaborative effort involving many contributors under Gatterer's supervision, it is perhaps unsurprising that the information printed in 1755 was compiled from many sources. In the last case presented, the engraving was most likely made from the drawing enclosed in the group of transcriptions predominantly penned by Karl Sigmund and not the original charter. This can be inferred from both the position of the parchment tags—or other flexible material—from which the seals hang and the seals’ stage of preservation. What is certain, though, is the fact that the drawing is larger than the engraving and features different line breaks. In a similar fashion, another reproduction displayed on the same plate does not present the width of the charter it depicts, an issue Gatterer would later criticize after he became a professor of History at the University of Göttingen. However, since in the particular case of the charter from March 15, 1263—and also the case with a second charter from December 16, 1366—the originals are still extant, they open up a window through which one may further investigate how historical evidence was produced and reproduced, not only in the context of an eighteenth‐century editorial enterprise but also within the records of the Holzschuher family and beyond.

## 5 Evidentiary Authority as a System

At the end of the eighteenth century, inaccuracies concerning the overall external appearance of engraved medieval charters drove Gatterer, as we have seen, to critically evaluate the plates included in Bessel's *Chronicon Gotwicense*, ultimately judging them useless for scientific purposes. However, not all reproductions of historical documents were made to meet the expectations of an eighteenth‐century auxiliary science of history. For example, Karl Sigmund commissioned engravings to illustrate the genealogical history of the Holzschuhers and help enlarge his family's reputation.^64^ For this reason, both the engravings printed in the book and the drawings featured in the Holzschuhers’ manuscript collections fulfilled, first and foremost, social expectations. What emerges prominently in the plates commissioned in the 1750s, as well as in the earlier handwritten bifolia, is the testimonial force of artifacts and their ability to affirm the ancient and noble origins of the family. Nonetheless, not all of the images inserted in the book published in 1755 would be considered useless for scientific purposes according to the scientific principles of diplomatics that Gatterer accepted at the end of his academic career.^65^

**Figure 11:**
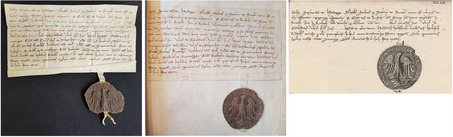
On the left—Original charter from 1263, March 15. Staatsarchiv Nürnberg, StAN Reichsstadt Nürnberg, Urkunden vor 1401 Nr. 33. At the center—Drawing: *Anhang zur Holzschuherischen Historie […]*. Historisches Archiv des Germanischen Nationalmuseums, Nürnberg, RS‐NBG 18‐Holzschuher‐15. On the right—Metal engraving. Detail of Plate XIII: Gatterer 1755. SUB Göttingen, 2 H BAV II 3102.

The drawing of the charter dated from 1263 preserves the overall external appearance of the document depicted, including its exact measurements (**Figure** **11**). The engraving of the same thirteenth‐century document, however, displays its textual content in excerpted form, giving the readers of the book published in 1755 a misleading idea about the external appearance of the particular historical artifact it depicts. Rather than being produced from the original, the engraving was more likely made from the drawing preserved together with further transcriptions of historical documents penned by Karl Sigmund Holzschuher.^66^ This was also the case with previous charters and, once more, suggested by the position of the parchment tag from which the seal hangs and its stage of preservation. In these cases, both the precise measurements of the historical artifact and its textual content were compressed to fit the engraved plate.

**Figure 12:**
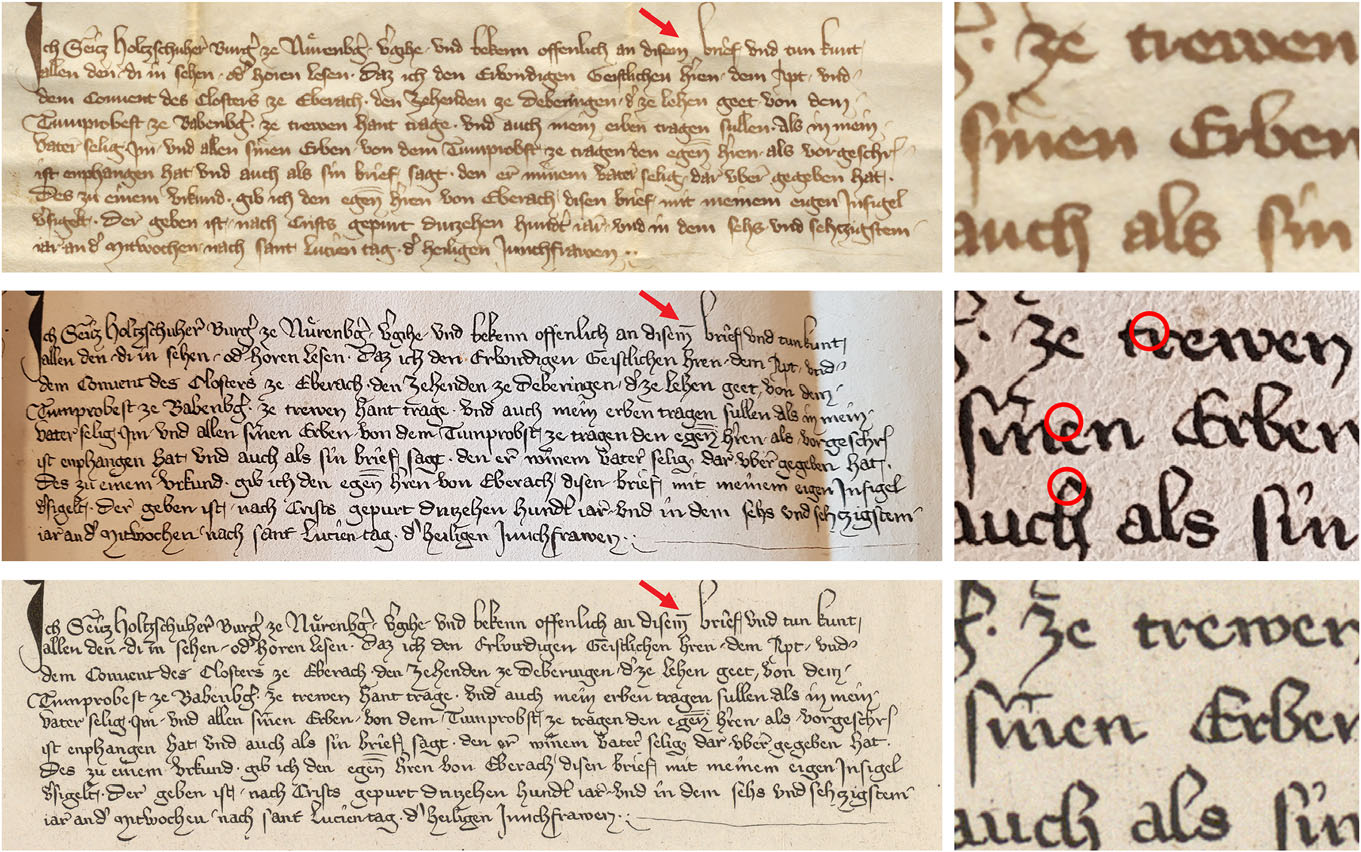
Above—Original charter: 1366, December 16. Staatsarchiv Würzburg, StAWü, Kloster Ebrach Urkunden 658. Middle—Drawing: *Anhang zur Holzschuherischen Historie […]*. Historisches Archiv des Germanischen Nationalmuseums, Nürnberg, RS‐NBG 18‐Holzschuher‐15. Below—Metal engraving. Detail of Plate XIV: Gatterer 1755. SUB Göttingen, 2 H BAV II 3102.

Tracking how information was compiled and (re)produced across the numerous manuscript copies of historical artifacts gathered by the Holzschuhers is a complex endeavor. Within the same collection of documents assembled by Karl Sigmund, there is also a drawing of an additional medieval charter reproduced on plate XIV. Although the historical information was rendered on paper in ink, the quill traced over an underlying pencil draft (**Figure** **12**). However, it remains challenging to determine whether the information in pencil directly followed the original charter. What is certain is that, considering the form of the diacritical marks placed above some letters—or even the absence of a diacritical mark in the original chart over the word *di[e]sem* in the first line—the drawing and the metal engraving show striking similarities.

When reproducing the charters displayed on plates XIII and XIV, Paul Küffner used either a quill or a needle in a waxed ground to etch their textual content. In contrast, the burin was preferred for engraving the hanging seals directly on the metal plates. The engraving was likely produced after the drawing, given the similarities between the two pieces. Regardless of the order that can be established in this chain of visual information, the choices made by the engraver are clear evidence of his intention to present the external appearance of the medieval artifacts he was commissioned to depict in a different medium.

As shown in the previous sections, many techniques were used to produce and reproduce historical information on parchment, wood, metal, and paper throughout the centuries. Moreover, there were many handwritten, woven, carved, painted, and drawn sources from which the *Historia genealogica,* printed in 1755, was assembled. In their personal archives, patrician families of German Imperial Cities—such as the Holzschuhers—employed various hands and methods from a very early date to store information related to their noble present and past. Faced with the threat that the product of those hands and methods might produce and reproduce inaccurate records, Gatterer developed a handmade reference system on paper through which the historical information related to the genealogical history of the Holzschuher family could be checked, thus securing authoritative historical knowledge. As Markus Friedrich has recently argued, the differences between many “sites of genealogical knowledge production and the epistemic standards and knowledge practices that shaped them” were significant.^67^ These differences also apply to further auxiliary sciences of history.

Seen from the perspective of Gatterer's classroom when the eighteenth century drew to a close, some of the plates engraved for the illustrated book that had motivated his hiring as a professor of History at the University of Göttingen at the end of the 1750s might have appeared useless for the study of diplomatics. Nevertheless, by reconstructing the multiple production stages of this editorial enterprise, I have tried to demonstrate that both the textual and the visual content of the work the young Gatterer was commissioned to write relied on an information system based on the interplay between verbal and visual information and, in turn, their relationship to the (re)produced material evidence of the past. Within this framework, evidentiary authority was ensured not by the engraved plates themselves but by a system shaped by the different media through which this system itself was produced, on the one hand, and the historical evidence which it reproduced, on the other. Delving into the knowledge‐making practices that underpinned Gatterer's editorial enterprise provides an illustrative example of how historical knowledge in the eighteenth century was achieved through complex scholarly, artistic, and editorial negotiations that encompassed graphic and intellectual authorship, intellectual authority, and disputes that occurred both in the making of visual evidence and in the trading of authoritative editions. As I have argued, the hands behind those efforts were not always empirical testimonies to the original artifacts they aimed to describe or depict. Instead, they are historical evidence of a system that relied on large chains of information, one characteristic of early modern collective^68^ and cumulative empiricism.

## Conflict of Interest

The authors declare no conflict of interest.

## Data Availability

Data sharing not applicable to this article as no datasets were generated or analyzed during the current study.

## Footnotes

1 Gierl 2012, on 1–3.

2 Gatterer 1799, on 175.

3 Quoted after: Rabe 2017, on 248.

4 Gatterer 1755, Henceforth: *Historia genealogica*.

5 “Brief von Christoph Sigmund von Holzschuher (Nürnberg, 16. Octob. 1755).” Historisches Archiv des Germanischen Nationalmuseums, Nürnberg, Nachlässe: Oetter, Samuel Wilhem, 104.

6 “Extr. Prot. d. d. 19. May 1752.” Stadtarchiv Nürnberg, E 49/II Nr. 628.

7 Gatterer 1767, on 35.

8 See: Bauer 2013, on 118.

9 Gatterer 1755, on a2^v^.

10 Gatterer 1752, on (3^v^–)(4^r^).

11 “d. d. 18. May 1753.” Stadtarchiv Nürnberg, E 49/II Nr. 628.

12 Gatterer 1799, on 175.

13 See: Schnabel 1992–1993, on 78.

14 Gatterer 1799, on 102.

15 Mulsow 2017, on 262.

16 Daston 2015, on 17.

17 Heumann 1745.

18 Mabillon 1681.

19 See: Woolf 2011, on 202–203; Momigliano 1990, on 73.

20 Grafton 2020, on 93.

21 Ibid., on 80 and 89, respectively.

22 See: Heumann 1745, on 8.

23 Gatterer 1798.

24 Gatterer 1765, on 149.

25 Montfaucon 1757.

26 Ibid., “Vorrede.” As a matter of fact, some of the engravings that figured in Montfaucon's volumes were copies of older ones. See: Miller 2012, on 372–373; Zwink 2006, on 163.

27 Gatterer 1755, on h2^r^.

28 Wood 2008, on 36.

29 See: Gatterer 1755, on h2^r^.

30 See: Georg Daniel Heumann, *Wahre Abbildung der […] Stadt Göttingen*, Göttingen, 1748–1750. SUB Göttingen, GR 2 HLP IV, 68/10 RARA, f. 77.

31 Daston and Galison 2007, on 81.

32 See: Steinke 2005.

33 Haller 1745.

34 Appel 2022, on 476.

35 Daston 2015, on 19.

36 See: Haller 1758.

37 Smellie 1758.

38 Seligmann 1748.

39 Gatterer 1755, on i1^v^.

40 Ibid., on 260.

41 Gierl 2012, on 159.

42 Gatterer 1767, on 35.

43 See: Gatterer 1799, on 55–56.

44 Gatterer 1767, on 35.

45 Johannes Christophorus Gattererus, *Historia Genealogica Dominorum Holzschuherorum*, c. 1755. Bibliothek des Germanischen Nationalmuseums, Nürnberg, Hs 28 889.

46 “Extr. Prot. d. d. 31 May 1754.” Stadtarchiv Nürnberg, E 49/II Nr. 628.

47 Johannes Christophorus Gattererus, *Historia Genealogica Dominorum Holzschuherorum*, c. 1755. Bibliothek des Germanischen Nationalmuseums, Nürnberg, Hs 28 889, Fasc. 1–2.

48 For a sample of Gatterer's handwriting in the 1750s, see his autograph correspondences preserved at the Universitätsarchiv in Göttingen: SUB Göttingen, UniA GÖ, Kur. Pers., 5757.

49 See: Gatterer 1799, on 55–56. See also: Gierl 2012, on 157.

50 Eddy 2023, on 87.

51 Blair 2010, on 212. Lorraine Daston, Richard Yeo, and Matthew Daniel Eddy investigated such note‐taking strategies from the standpoint of scientific practices, and more recently, Elaine Leong 2018 framed them as an epistemic activity. See: Daston 2004; Yeo 2014; Eddy 2016; Leong 2018, on 80.

52 “Extr. Prot. d. d. 31 May 1754.” Stadtarchiv Nürnberg, E 49/II Nr. 628.

53 Friedrich 2023, on 4 and 70, respectively. For the legal significance of such documents within the Holy Roman Empire, see: Eskildsen, u^2022^, on 51.

54
*Geschlechterchronik der Nürnberger Familie Holzschuher, d.h. Verzeichnis aller Holzschuer seit Heinrich Holzschuher 1258. Angelegt von Lazarus Holzschuher (†1523) im Jahre 1505. Mit angehängtem Verzeichnis aller mannbaren Holzschuher, die im Jahre 1511 (an St. Egidientag) gelebt haben*, Staatsarchiv Nürnberg, Rst. Nürnberg, Handschrift 281 (168). See also: Hirschmann 1979, on 108.

55
*Allerley Alte Brieffe, Urkunden, und Stifftungen, Geschichte, und anders Hießige Gemeine Statt, und dero Geschlecht betreffendt: Aus allerley Wahrhaften Urkunden zusammen getragen von Veit Hieronimus Holzschuher. Anno 1693*, Stadtarchiv Nürnberg, E 49/III Nr. 94.

56
*Der Herren Holtzschuer Vr‐Alteß herkommen, Geschlecht und Stammen Beschreibung. Von Johann Herold. 1736*, Bibliothek des Germanischen Nationalmuseums, Nürnberg, Hs 28 890 and Hs 28 891.

57
*Sammlung von 190 Urkunden aus den Jahren 1228‐1588 zur Geschichte der Familie Holzschuher. Abschriften um 1750*, Historisches Archiv des Germanischen Nationalmuseums, Nürnberg, RS‐NBG 18‐Holzschuher‐19.

58
Historisches Archiv des Germanischen Nationalmuseums, Nürnberg, RS‐NBG 13‐Holzschuher‐22.

59
*Anhang zur Holzschuherischen Historie, bestehend aus verschiedenen Stiftungs‐ und anderen Briefen darauf man sich zu mehren Beweiß bezogen hat. Anno 1724*, Historisches Archiv des Germanischen Nationalmuseums, Nürnberg, RS‐NBG 18‐Holzschuher‐15.

60 Gatterer 1755, on k1^r^.

61 Albrecht 1761a; Albrecht 1761b; Albrecht 1764.

62 Grafton 2020, on 92.

63 Gatterer 1755, on c2^v^: “[…] uetusta rerum documenta, partim integra, partim excerpta […].”

64 “d. d. 18. May 1753.” Stadtarchiv Nürnberg, E 49/II Nr. 628.

65 Gatterer 1799.

66
*Anhang zur Holzschuherischen Historie […].* Historisches Archiv des Germanischen Nationalmuseums, Nürnberg, RS‐NBG 18‐Holzschuher‐15.

67 Friedrich 2023, on 204.

68 Daston 2012.
